# Customizable 3D printed perfusion bioreactor for the engineering of stem cell microenvironments

**DOI:** 10.3389/fbioe.2022.1081145

**Published:** 2023-01-09

**Authors:** Steven J. Dupard, Alejandro Garcia Garcia, Paul E. Bourgine

**Affiliations:** ^1^ Cell, Tissue and Organ engineering laboratory, Biomedical Centre (BMC), Department of Clinical Sciences Lund, Stem Cell Centre, Lund University, Lund, Sweden; ^2^ Wallenberg Centre for Molecular Medicine, Lund University, Lund, Sweden

**Keywords:** 3D printing, polylactic acid, bioreactor, mesenchymal niche, hematopoiesis, collagen scaffold, 3D culture

## Abstract

Faithful modeling of tissues and organs requires the development of systems reflecting their dynamic 3D cellular architecture and organization. Current technologies suffer from a lack of design flexibility and complex prototyping, preventing their broad adoption by the scientific community. To make 3D cell culture more available and adaptable we here describe the use of the fused deposition modeling (FDM) technology to rapid-prototype 3D printed perfusion bioreactors. Our 3D printed bioreactors are made of polylactic acid resulting in reusable systems customizable in size and shape. Following design confirmation, our bioreactors were biologically validated for the culture of human mesenchymal stromal cells under perfusion for up to 2 weeks on collagen scaffolds. Microenvironments of various size/volume (6–12 mm in diameter) could be engineered, by modulating the 3D printed bioreactor design. Metabolic assay and confocal microscopy confirmed the homogenous mesenchymal cell distribution throughout the material pores. The resulting human microenvironments were further exploited for the maintenance of human hematopoietic stem cells. Following 1 week of stromal coculture, we report the recapitulation of 3D interactions between the mesenchymal and hematopoietic fractions, associated with a phenotypic expansion of the blood stem cell populations.Our data confirm that perfusion bioreactors fit for cell culture can be generated using a 3D printing technology and exploited for the 3D modeling of complex stem cell systems. Our approach opens the gates for a more faithful investigation of cellular processes in relation to a dynamic 3D microenvironment.

## 1 Introduction

In the last decade, accumulated evidence about the importance of the stem cell microenvironment in modeling human tissue *in vitro* led to a transition from 2D to 3D culture (Caswell and Zech, 2018; Ingber, 2020; Jensen and Teng, 2020; Dudaryeva et al., 2021; Indana et al., 2021; Jacchetti et al., 2021; Berger et al., 2022). As compared to conventional planar models, 3D systems add a new layer of biological relevance by recapitulating 3D tissue complexity and structural organizations, including critical cell-to-cell and cell-matrix interactions (Birgersdotter et al., 2005; Anselme et al., 2018; Jensen and Teng, 2020; Dudaryeva et al., 2021; Yamada et al., 2022). Remarkably, this higher modeling power translated into superior maintenance of stem cell properties in a wide array of tissue (Frith et al., 2010; Huebsch et al., 2010; Li et al., 2016; Madl et al., 2017). Beyond new fundamental opportunities in tissue/organ modeling, 3D systems may help address the unmet need of replacing animal-based drug screening approaches. Indeed, the relevance of animal models and/or non-human *in vitro* systems is questionable for the development of human-tailored therapeutic strategies, including new biologics. Therefore, 3D systems are now regarded as promising middle grounds between animal models and human trials (Ingber, 2020; Jensen and Teng, 2020).

A myriad of 3D systems was developed in recent years, each bearing advantages and limitations in their biomimetic capacity (Jensen and Teng, 2020). Static culture of scaffolding materials (e.g., hydrogels, ceramics, titanium, decellularized matrices) were among the first methods providing a 3D cellular environment. While pioneering the investigation of 3D cues on cellular activity, these systems were limited by their reliance on the passive diffusion of nutrients and oxygen to establish a nurturing environment of superior dimension (Billiet et al., 2012; Ruedinger et al., 2015). Similarly, organoid protocols are size-restricted due to the tissue growth leaving a necrotic core, in addition to exhibiting limited cell responsiveness and batch-to-batch variability (Hofer and Lutolf, 2021). Delivery of nutrients and waste removal were improved *via* dynamic media perfusion approaches (Wendt et al., 2003). The use of perfusion bioreactors resolved the size limitation while offering another array of microenvironmental control, with fluid shear-stress and dynamic mechanical stimulation (Davisson et al., 2002; Powers et al., 2002; Song et al., 2005; Raimondi et al., 2006; Cioffi et al., 2008; McCoy and O’Brien, 2010). Despite their superior biological relevance, perfusion bioreactors lack the affordability, ease of use, and scalability of 2D systems, which hinders their broader adoption. To circumvent these constraints, 3D printing techniques can be harnessed for the creation of 3D culture devices.

We here aim at developing a customizable 3D perfusion bioreactor system by exploiting a 3D printing approach. Our strategy relies on the use of fused deposition modeling (FDM) of polylactic acid (PLA) filament to enable the rapid generation and modification of the perfusion chamber homing the 3D culture. We focused on the establishment of stromal niches of variable dimensions to highlight the adaptability of our system. Last, we target the biological validation of the 3D perfusion bioreactor by engineering human bone marrow hematopoietic niches. The foundation of this microenvironment lies within the interactions between human mesenchymal stromal cells (hMSCs) and hematopoietic stem cells (HSCs) indispensable to the healthy hematopoietic process in the bone marrow (Mendelson and Frenette, 2014; Wei and Frenette, 2018).

## 2 Results

### 2.1 Design validation and disinfection of 3D printed bioreactor systems.

**FIGURE 1:**
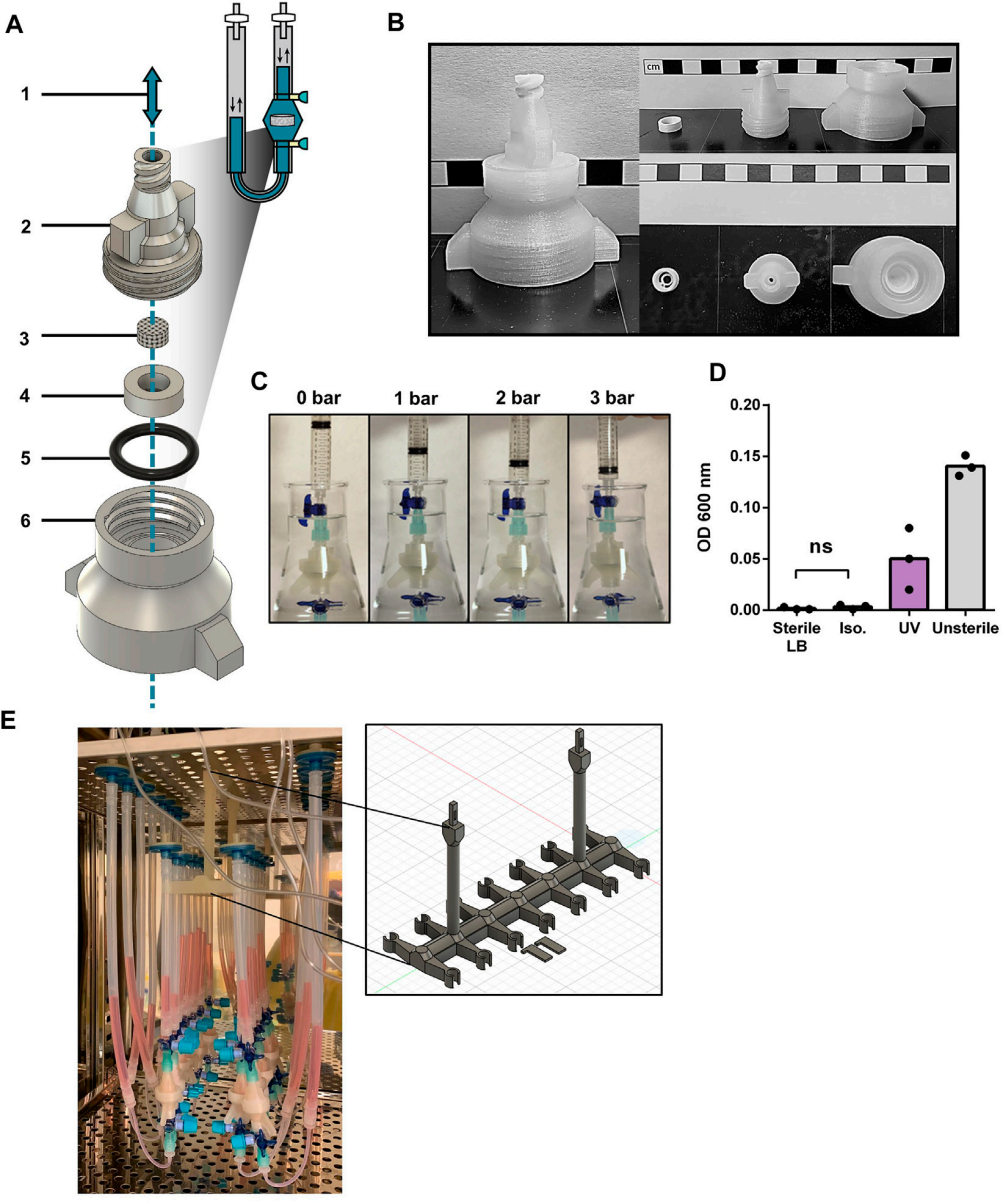
Design validation and disinfection of 3D printed bioreactor systems **(A)** Diagram of the chamber components of the 3D printed perfusion bioreactor. 1: Representation of the alternating perfusion of culture media (blue) through the bioreactor; 2: Upper stage of the bioreactor chamber; 3: Collagen I (Col1) scaffold; 4: Scaffold holder; 5: Silicon O-ring; 6: Lower stage of the bioreactor chamber. Components 2, 4, and 6 are 3D printed with polylactic acid (PLA), components 3 and 5 are commercially available. **(B)** Printed components 2, 4, and 6 of the perfusion bioreactor. Scale bars represent 1 cm. **(C)** Airtightness assessment of the mounted bioreactor chamber by water submersion and increased pressure exposure. **(D)** Nanodrop OD600 of lysogeny broth (LB) media incubated with PLA bioreactor components overnight at 37 °C, after 30 min exposure to UV, 70% Isopropanol (Iso.) or PBS (Unsterile). Unpaired t-test (n = 3). Ns = *p*-value >.05. **(E)** Photograph of an array of 14 3D printed bioreactors in use within a cell culture incubator. The framed picture highlights the 3D printed PLA bioreactor holder used during culture.

We here describe the design of a 3D printed oscillating perfusion bioreactor, offering bidirectional fluid circulation in a cyclic manner (Supplementary Video S1). Our bioreactor chamber is composed of printed parts (Figures 1A, B and Supplementary Figure S1A) designed in Fusion360™ and printed in 1 h with a standard biodegradable bioplastic PLA (Castro-Aguirre et al., 2016) using a cost-effective Prusa™ MK3 printer. The printed parts consist of a scaffold holder and the upper and lower components of the perfusion chamber. The chamber components interlock through a thread to encapsulate the scaffolding material held by the scaffold holder. This junction is reinforced with a silicon O-ring. At both ends of the chamber, 3-way stopcocks create syringe-accessible links with the tubing. The later carries the media which oscillate through the chamber *via* a programmable pump. To prevent contamination, the system has two filters at both tubing ends, and disinfectant caps on the stopcock valves.

The design was fashion to accommodates the printing process (no hard curves or overhangs that necessitate support material) while supporting fluidic and airtightness (random seams placement, additional perimeters, grove and threads for tubing and silicon O-ring). Printing parameters were also optimized to favor printed layer-to-layer adhesion (listed in Supplementary Table S1). We also incorporated in the design a thread fitting 50 mL tubes at the bottom of the chamber, towards facilitating sample collection at the end of a culture process (Supplementary Figure S1B). Airtightness of printed chambers was first tested by gradual air pressure application for up to 3 bar (Figure 1C). No detectable leakage was observed during repeated test with a pool of 15 printed bioreactors. While having a better layer adhesion compared to autoclavable FDM filaments such as polypropylene and nylon, PLA cannot be autoclaved as its glass transition temperature is reached at 50°C. Hence, alcohol bath and UV exposure were investigated as other means of disinfection. Alcohol disinfection was performed with a 30 min immersion in 70% isopropanol (Figure 1D), previously reported to not degrade 3D printed PLA (Erokhin et al., 2019). UV light disinfection consisted in 30 min of exposure using a standard benchtop UV cabinet. Following treatment, disinfection was assessed by placing respective chambers for 24 h in flasks containing lysogeny broth (LB) at 37°C overnight under agitation. The growth of bacteria was assessed by optical density at 600 nm. Only the disinfection by isopropanol prevented bacteria growth, as opposed to UV light which did not reach the same level of disinfection. In addition, no mycoplasma contamination could be detected during culture (three experimental repeats, data not shown).

We thus here validate the design, printing, and disinfection of perfusion bioreactor chambers. In addition, we further developed a 3D printed bioreactor holder for placement in incubators (Figure 1E), as well as a bioreactor stand (Supplementary Figure S1C) for bench manipulation of the system. This offers easy handling and optimization of space for multiple bioreactor experiments.

### 2.2 Stromal environment of tailored dimensions can be established within 3D printed perfusion bioreactors

**FIGURE 2:**
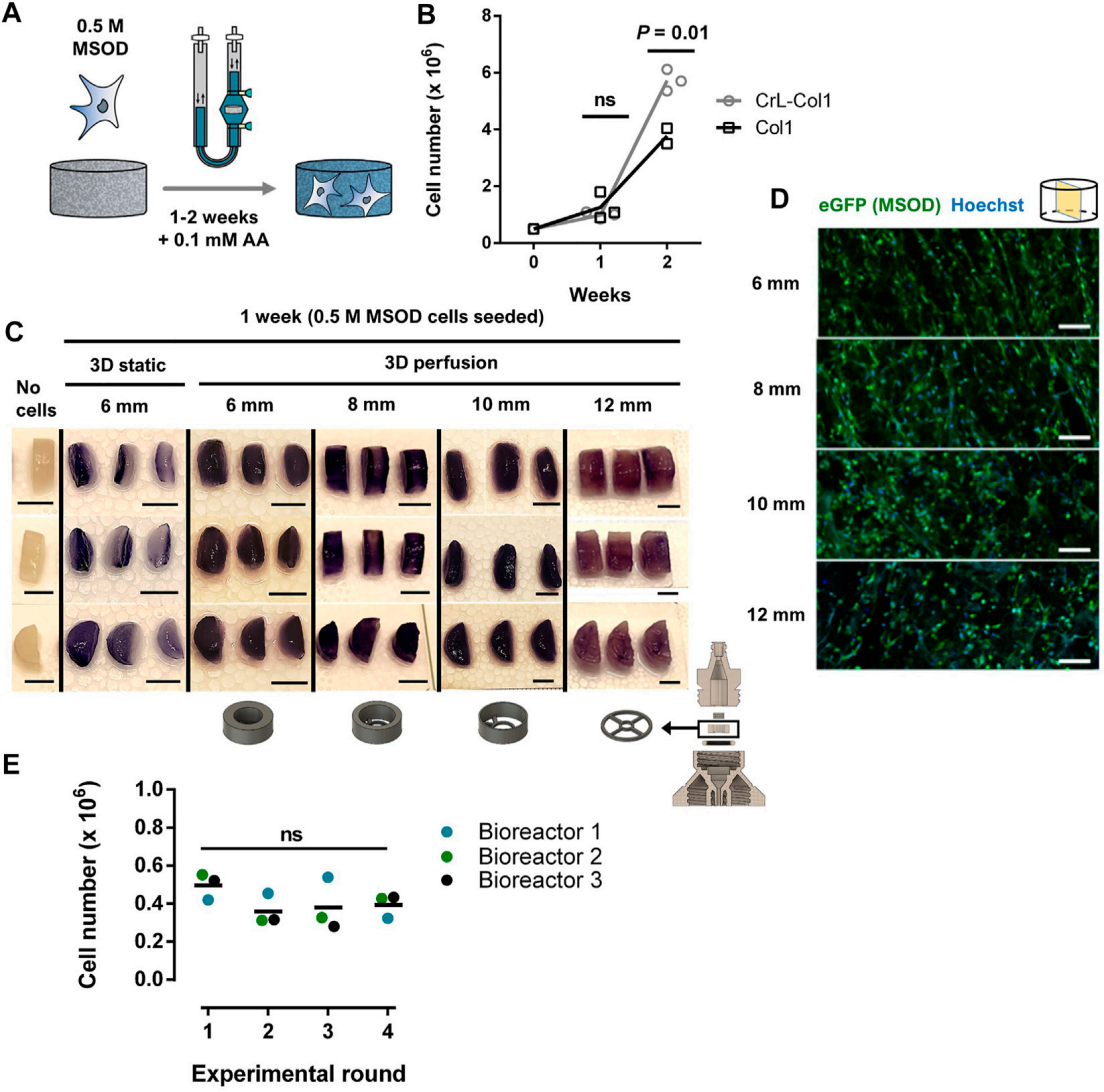
Stromal environments of tailored dimensions can be established within 3D printed perfusion bioreactors. **(A)** Experimental diagram of the MSOD culture within the 3D printed perfusion bioreactor. Cells are dynamically cultured in the system for up to 2 weeks. AA: Ascorbic Acid. **(B)** MSOD cell number quantification (CyQuant™ DNA) on collagen 1 (Col1) and crosslinked (CrL)-Col1 scaffolds culture in perfusion bioreactor over a 2 weeks time course. Unpaired t-test (n = 3). Ns = *p*-value >.05. **(C)** MTT assay for assessment of the cellular metabolic activity within the 6–12 mm diameter CrL-Col1 scaffolds following 1 week of static/perfusion bioreactor culture. The scaffold holder size was adjusted (bottom) to the size of the scaffold. In static condition, the scaffolds were seeded and maintained in a 12-well plate. The scaffold was cut through the median plane for better visualization of the scaffold core (n = 3). Scale bar = 5 mm. **(D)** Confocal microscopy analysis of six up to 12 mm CrL-Col1 scaffolds cross-section (median plane), evidencing the homogenous MSOD cells (eGFP positive) distribution after 1 week of culture. N = 3. Scale = 100 μm eGFP: enhanced green fluorescent protein. **(E)** MSOD cell number quantification (CyQuant™ DNA) following 3 days of culture in reused bioreactors. One-way ANOVA (n = 3). Ns = *p*-value >.05.

We next aimed at validating our 3D printed system for the culture of human cells, towards the engineering of 3D microenvironments. To this end, we exploited a pre-established human mesenchymal stromal cell line (MSOD, Mesenchymal Sword Of Damocles) (Bourgine et al., 2014) (Supplementary Figure S2A). The MSOD line offers unlimited cell supply and standardization while maintaining properties of primary bone-marrow hMSCs. MSOD cells were dynamically seeded overnight on a collagen type I scaffold (Col1) and cultured for up to 2 weeks (Figure 2A). Collagen scaffolds have proven to be versatile materials thus exploitable in multiple organ/tissue modelling strategies (Meinel et al., 2004; Liu et al., 2009; Zhou et al., 2011; Ding et al., 2014; Chan et al., 2016). To assess cell colonization potential in long term culture, we perform a comparison between a Col1 and an alternative hexamethylene diisocyanate crosslinked collagen scaffold (CrL-Col1) within our system (Figure 2B, Supplementary Figure S2B–D,E). At 1 week of culture, both Col1 and CrL-Col1 scaffolds contained a 2-fold increase in cell number from input (5 million MSOD). However, at 2 weeks, an 8-fold increase was observed for Col1 while the CrL-Col1 scaffold presented a 12-fold increase. This disparity can be explained by the degradation of the Col1 scaffold, evident after 2 weeks of culture compared to CrL-Col1 (Supplementary Figure S2B).

Based on these results, the CrL-Col1 was thus selected for the rest of the study. We further characterized the porosity and provided previsously determined mechanical properties of our selected material (Supplementary Figure S2D, E, (Chaudhury et al., 2012)), revealing an interconnected pore network mainly comprised in the 50–100 µm range (>60%). Next, we demonstrated the versatility of the 3D printing approach towards the engineering of stromal environment of different dimensions. Bioreactor chambers capable of hosting CrL-Col1 scaffold of 6, 8, 10, and 12 mm were designed, and MSOD cells were cultured in the respective systems for a week. In contrast to the superficial scaffold colonization observed in 3D static culture (Figure 2C), MTT assay revealed the homogenous MSOD cell distribution within the scaffolding materials under dynamic perfusion culture (Figure 2C). This uniform distribution was further confirmed by confocal microscopy, with presence of MSOD cells detected across the CrL-Col1 material (Figure 2D) independently of the tissue size (Supplementary Figure S2C). This uniformity is crucial to ensure the functionalization of the scaffold with the supportive ability of the stromal cells, analogous to their role in the bone marrow tissue (Klimczak and Kozlowska, 2016).

We further assessed the capacity of removing proteins from 3D printed parts post-culture, towards demonstrating its reusability. To this end, we quantified protein content on the scaffold holder, as the bioreactor part primarily in contact with biological material (Supplementary Figure S2F). Prior to cleaning, scaffold holders carried 303.49 ± 70.79 µg of protein, while content on cleaned and freshly printed scaffold holders could not be detected (<20 µg). We next performed 4 successive rounds of 3D perfusion culture using the same 3 distinct bioreactors (Figure 2E), cleaned prior to each new experiment. We report a similar MSOD cell growth independently of the experimental round (CyQuant^TM^ quantification, Figure 2E), thus demonstrating the reusability of our 3D system.

### 2.3 Stromal environment engineered in 3D printed bioreactors results in superior human hematopoietic stem cell maintenance

**FIGURE 3:**
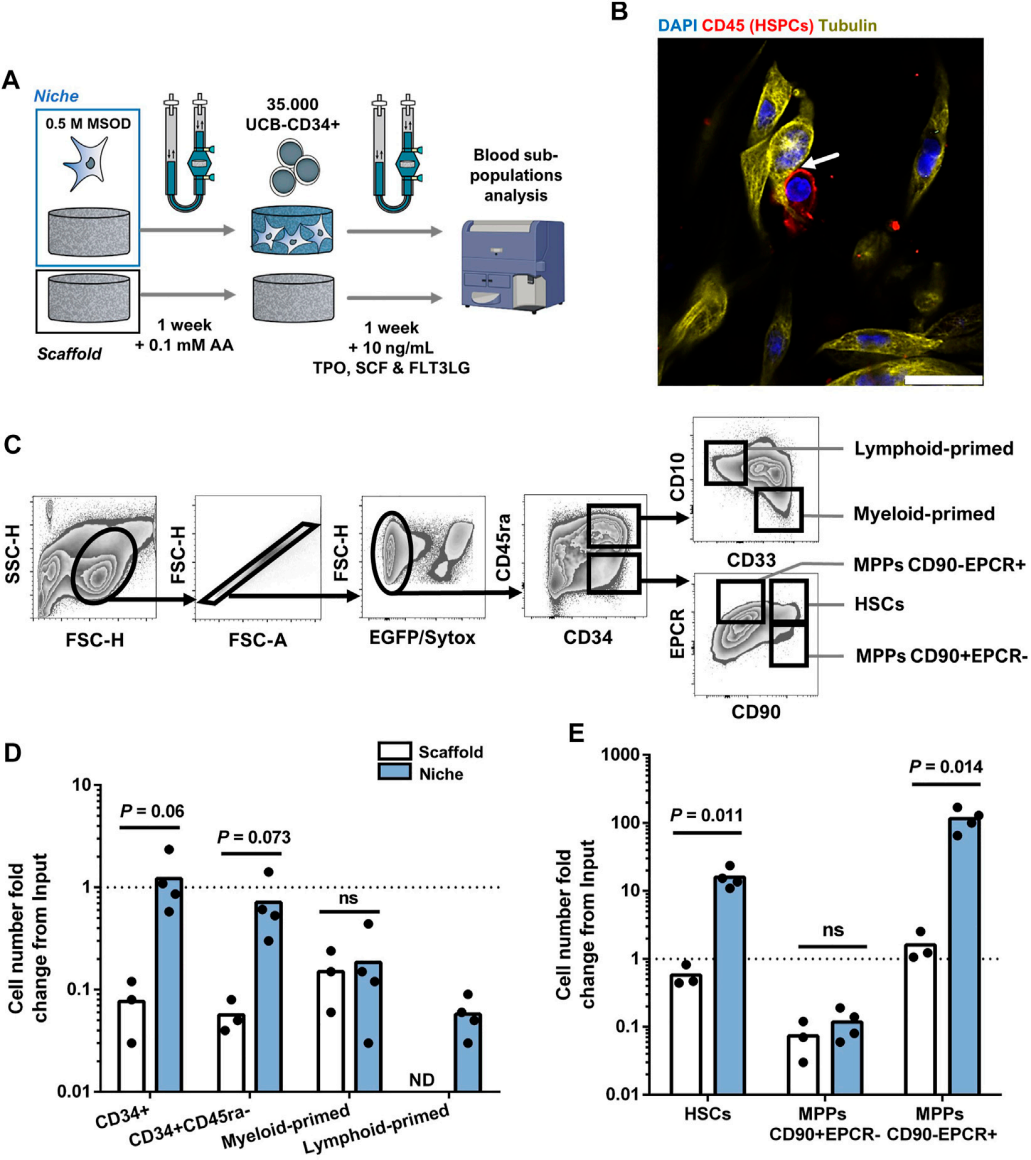
Stromal environment engineered in 3D printed bioreactors results in superior human hematopoietic stem cell maintenance. **(A)** Experimental scheme of the 3D coculture using MSOD and UCB-CD34^+^ cells. Briefly, UCB-CD34^+^ cells were cultured in an empty scaffold (Scaffold) or an engineered MSOD niche (Niche) for a week. Blood populations were subsequently retrieved for quantitative phenotypic analysis. AA: Ascorbic Acid; TPO: Thrombopoietin; SCF: Stem Cell Factor (also known as Kit ligand); FLT3LG: Fms-related tyrosine kinase three ligand. **(B)** Confocal microscopy picture of the engineered Niche 2 days after the addition of UCB-CD34^+^ cells. Physical interactions between the mesenchymal (MSOD) and blood compartments (CD45) could be identified (arrow). Tubulin (Cy3; Yellow) delineates both MSOD and HSPCs while CD45 (CF633; Red) identifies blood cells only. DAPI stains nuclei (blue). Scale = 20 µm. **(C)** Flow cytometry gating strategy used to identify mesenchymal and hematopoietic populations. **(D)** and **(E)** respectively show hematopoietic committed and stem populations fold change from the UCB-CD34^+^ input (dotted line) at the end of the culture. The “Scaffold” condition refers to maintenance in culture without stromal cells, as opposed to the “Niche” condition. A significant expansion of the stem populations (HSCs and MPPs CD90-/EPCR+) could be observed in the Niche condition. Unpaired t-test (n ≥ 3). Ns = *p*-value >.1. ND: Not Detected; HSCs: Hematopoietic Stem Cells; MPPs: Multipotent Progenitors.

Mesenchymal cells are an important component of the bone marrow niche, supporting the function of hematopoietic stem cells. Modeling the human BM niche *ex vivo* remains challenging, since HSCs can hardly be maintained in standard *in vitro* conditions (Kumar and Geiger, 2017). Here, we aimed at assessing the suitability of our 3D printed bioreactor for the engineering of BM microenvironment sustaining HSCs survival. To this end, MSOD cells were first cultured in our perfusion bioreactor for a week to functionalize the CrL-Col1 scaffold (6 mm). Subsequently, primary human CD34^+^ hematopoietic cells from cord-blood origin were added to the bioreactor, and cocultured in presence of low concentration of hematopoietic cytokines (Figure 3A). Within the engineered tissue (Supplementary Figure S3A), physical interactions between the mesenchymal fraction and hematopoietic cells (CD45^+^) could be evidenced using staining for the cytoskeletal protein tubulin (Figure 3B). Via confocal microscopy, a 3D stack of the established bone marrow microenvironment was reconstructed which not only further identified hMSCs-HSPCs interactions, but also highlighted the complex networks of intertwined hMSCs cytoplasmic protrusions (Supplementary Video S2, white arrows highlight interactions). After a week of coculture in a 3D printed bioreactor, the hematopoietic cells were harvested for quantitative phenotypic analysis by flow cytometry.

Tissues were recovered from the chamber and digested for cell retrieval, prior to immune staining for a panel of phenotypic markers indicating the lineage commitment, or absence thereof, within the hematopoietic CD34^+^ subpopulations (Figure 3C). Briefly, we first excluded MSOD, and dead cells based on positivity to GFP and Sytox Green. The negative fraction containing CD34^+^ cells was then further characterized for stem or commitment phenotypes (see Material and Methods section). As a 3D control, we similarly assessed the survival and development of blood cells cultured on the material only (“Scaffold”), thus deprived of MSOD cells.

As compared to the “Scaffold” condition, the number of CD34^+^ HSPCs retrieved from the “Niche” engineered tissue remained stable over the 7 days of coculture (18.06 ± 13.39 fold decrease in Scaffold against 1.22 ± .782 fold increase in Niche condition; Figure 3D). Yet, we observed a general decrease in the number of more committed myeloid-primed (CD34^+^CD45ra+CD10^−^CD33^+^) and lymphoid-primed (CD34^+^CD45ra+CD10^+^CD33^−^) progenitors in both the Scaffold and Niche settings (Figure 3D). The more stem CD34^+^CD45ra-population also decreased overtime in both conditions, though to a lower extend in the presence of MSOD (1.89 ± 1.09 fold decrease against 19.17 ± 6.29 in Scaffold). To further identify which stem populations were affected by this decline, we used the CD90 and EPCR markers to separate phenotypic HSCs (CD34^+^CD45ra-CD90+EPCR+) from the multipotent progenitors (MPPs) CD34^+^CD45ra-CD90+EPCR- and CD34^+^CD45ra-CD90-EPCR+ (Figure 3C). Importantly, MPPs EPCR+ were recently demonstrated to possess a higher MMPs re-population capacity (Fares et al., 2017; Anjos-Afonso et al., 2022).

We observed a stark difference in the stem subpopulation outputs during the 3D coculture between Niche and Scaffold conditions (Figure 3E). In both conditions, MPPs CD90+EPCR-drastically decreased as compared to the starting population, with a 18.65 (±13.05) fold decrease for Scaffold and 12.10 (±4.77) for Niche. However, while MPPs CD90-EPCR+ were maintained in Scaffold (1.61 ± .80 fold increase), we observe a 97.87 (±31.92) fold increase in presence of MSOD cells. Importantly, in contrast to Scaffold (1.873 ± .571 fold decrease), we measured a 13.30 (±2.25) fold increase in HSCs population when cocultured with MSOD cells. Remarkably, the observed hematopoietic support of MSOD in 3D coculture within our 3D printed perfusion bioreactor was not reflected in 2D culture (Supplementary Figure S3B), where MSOD does not exhibit striking hematopoietic support as compared to a no-stroma condition (Supplementary Figure SC-D).

Altogether, our data validate the generation of human bone marrow niche using a 3D perfusion bioreactor. In contrast to 2D culture and non-stromal conditions, we report superior maintenance of phenotypic HSCs and MPPs with high-repopulation potential on engineered microenvironments.

## 3 Discussion

In the present study, we report a reusable 3D printed perfusion bioreactor fit for culture of adherent and non-adherent human stem cells. We demonstrated fast prototyping of various bioreactor sizes, offering the engineering of tissue with tailored dimension. Our system was validated for the generation of human bone marrow proxy, whereby the 3D environment and hMSC-HSPCs interactions resulted in the superior maintenance/expansion of phenotypic HSCs.

Oscillating perfusion bioreactors present multiple advantages for 3D cell culture but suffer from rigid designs and demanding resources. By choosing PLA as a building material coupled to an inexpensive printer, we obtained an open source designed bioreactor that is cost-efficient (less than 1€ per bioreactor). PLA, while being inexpensive, is also routinely used as biocompatible medical screws, rods, and plates (Middleton and Tipton, 2000; da Silva et al., 2018); and even for facial esthetic interventions (Schierle and Casas, 2011). Importantly, 3D printed PLA does not present any adverse effects on the viability of primary human cells either in direct or indirect culture (Schmelzer et al., 2016).

We optimized the printing parameters to enhance the layer-to-layer adhesion with the aim to bring airtightness at printing, preventing time-consuming post-processing. We envision that the insights gathered about the printing process could encourage other investigators to develop, prototype and manufacture their own methods for cell culture or liquid handling systems. In contrast to rigid devices of commercially available alternatives, design/dimension of the system can be rapidly tailored to the experimental needs, towards miniaturization of the system or scaled-up generation of tissues. This would reveal practical in order to parallelized multiple conditions in micro-bioreactor systems, or conversely target the engineering of larger tissue providing superior cellular throughput for multiple readouts.

Pre-existing 3D printed bioreactor devices exploit two main rapidly evolving technologies, FDM (also termed fused filament fabrication (FFF)) and resin-based stereolithography (SLA). Printing precision and material toxicity are primordial considerations for biologically compatible systems. SLA can achieve high precision in the printed construct, but solvents used present a toxicity for both users and cells (Zhu et al., 2015; MacDonald et al., 2016). In addition, available SLA printed bioreactors required post-processing (e.g. curing with UV light) prior to use (Anderson et al., 2013; Wilkinson et al., 2020; Gabetti et al., 2022). In parallel, FDM-printed devices relies on inexpensive plastic filaments already used in 2D cell culture but suffers from the lack of adaptability and reusability (Costa et al., 2015; Janvier et al., 2020) as well as limited biological validation (Schmid et al., 2018). Moreover, most available devices are embedded within a continuous media flow, which complicates their uses with non-adherent cells. Instead, our reusable system was validated for long-term culture, compatible with various scaffolding material in oscillating dynamic perfusion with primary human cells. This includes human HSCs, which survival and stem cell phenotype remain challenging to maintain *ex vivo*.

HSCs rely on their niche for both functional maintenance and survival (Scadden, 2006; Jones and Wagers, 2008). HSCs are notorious for rapidly losing their regenerative potential *ex vivo* (Kumar and Geiger, 2017; Wilkinson et al., 2020), which has prompted extensive research in reconstituting aspects of the HSC niche towards maintenance of HSCs properties (Bourgine et al., 2018a). MSCs are an essential constituent of the HSCs niche (Méndez-Ferrer et al., 2010) and as such were successfully employed as *ex vivo* HSCs support (Jing et al., 2010; Bourgine et al., 2018b). However, primary hMSCs sources exhibit variable differentiation potential and rapidly acquire senescence upon culture (Pevsner-Fischer et al., 2011; Yang et al., 2018). Here, we exploit MSOD cells as a standardized hMSCs source for HSPCs support in a 3D perfusion environment. MSOD retains the capacity to differentiate in various mesenchymal lineages, thus appearing as a potent cellular tool to decipher niche ontology in a reproducible manner (Bourgine et al., 2014; Pigeot et al., 2021). Furthermore, its stability would also enable further genetic modifications to direct the expression of hematopoietic factors and custom genetic cassettes for human-specific knowledge acquisition.

While this study does not aim at demonstrating the superiority of 3D dynamic over static cultures, the discrepancy of MSOD hematopoietic support in 2D and 3D culture may indicate the importance of the microenvironment for hematopoietic support. Indeed, previous work has demonstrated the decreased secretion of inflammatory cytokines and increased secretion of supportive hematopoietic factors by hMSCs in a dynamic (Diaz et al., 2017) and 3D microenvironment (Ylöstalo et al., 2012). In line with these studies, our data suggests that MSOD cells supportive capacity is improved in a 3D dynamic setting (Hoggatt et al., 2009; Mirantes et al., 2014). Altogether, understanding the molecular interaction between niche cells and HSCs within our 3D printed bioreactor could also inform the mechanisms leading to increased maintenance and expansion, ultimately benefiting transplantation and gene editing therapies (Wilkinson et al., 2020).

Beyond providing a novel platform for modeling normal and pathological hematopoiesis, the tools presented here could also be applied to other fields benefiting from a more democratized 3D culture system such as cardiac (Radisic et al., 2008), liver (Dash et al., 2009), and lung (Petersen et al., 2011) microenvironment engineering. The combination of our 3D printing perfusion bioreactor and MSOD can lead to the modeling of various tissue/organ composed of a stromal compartment (e.g., mammary glands, prostate and gut) and associated stem cells.

## 4 Conclusion

Our study proposes and validates the design of 3D printed bioreactors for the custom engineering of stem cell microenvironments. Such devices will facilitate and prompt the advanced modeling of normal or pathological stem cell processes, through the 3D recapitulation of complex tissue and organ systems.

## 5 Materials and methods

### 5.1 Ethic statement

Experimental work carried out with primary human samples was approved by the regional and ethical committee for Lund/Malmö (Regionala Etikprövningsnämnden I Lund/Malmö), approval no. 2010-695. Informed consent was obtained from mothers of the umbilical cord blood (UBC) donors, and all samples were de-identified before use in the present study.

### 5.2 Umbilical cord blood (UCB)-CD34^+^ cells isolation

UBC was collected at Skåne University Hospitals and Helsingborg Hospital. Briefly, mononuclear cells were collected by Ficoll separation and CD34^+^ cells were isolated using the CD34 MicroBead kit (Miltenyi Biotec #130-702) according to the manufacturer’s instructions. UCB-CD34^+^ cells samples used in this study were originating from a pool of a minimum of 3 units, processed within 24 h after collection and with a minimum CD34^+^ purity of 94% and viability of at least 95%.

### 5.3 Mesenchymal-hematopoietic 2D coculture

2D coculture was carried out in a Nucleon™ Delta Surface 12-well plate (ThermoFisher #140675). Briefly, 10.000 MSOD cells were cultured for 7 days in complete media (CM) composed of 500 mL of MEM-α (Gibco # 22571038) supplemented with 50 mL of tetracycline-free fetal bovine serum (FBS; ThermoFisher); 5 mL of sodium pyruvate (100 mM; Gibco #11360070); 5 mL of HEPES (1 M; Gibco # 15630080); 5 mL of Penicillin-Streptomycin-Glutamine (100x at 50 mg/mL; Gibco #10378016). Ascorbic acid (AA; 100 μM; Sigma #A8960-5G) was added at each media change which was performed every third day. On day 7, 4 Gy irradiation was performed using the CellRad X-ray source by Flaxitron, cells were then le ft to recover for another 24 h in fresh CM. 35.000 UCB-CD34^+^ cells were added in each well in 1 mL of Coculture media (CoM), composed of DMEM (High Glucose, no glutamine, no calcium; ThermoFisher #21068028); 20% BIT9500 Serum substitute (StemCellTechnologies #09500), 1% Penicillin-Streptomycin-Glutamine and 1% HEPES. Media change was performed every 2 days with the addition of .02% ß-mercaptoethanol (500X at 50 mM; ThermoFisher #31350010), human stem cell factor (SCF), thrombopoietin (TPO) and Fms-related tyrosine kinase 3 ligand (FLT3LG) at 10 ng/mL (all from Miltenyi Biotec, respectively #130-096-692, #130-095-745 and # 130-096-474) reconstituted in IMDM +10% Bovine Serum Albumin (StemCellTechnology #09300).

### 5.4 Bioreactor design and fused deposition modeling (FDM) 3D printing

The design of the bioreactor chambers (See Figure 1A; Supplementary Figure S1A) was modelized using Fusion 360 from Autodesk and sliced using PrusaSlicer 2.4 (Prusa) for a .4 mm nozzle and .15 mm layer height (see full details and critical parameters in Supplementary Table S1). Once sliced, the model was loaded to a Prusa i3 MK3S+ and printed with a polylactic acid (PLA) 1.75 mm ± .03 mm natural/transparent filament (Verbatim #55317) on a steel bed coated with UHU Twist and Glue without solvent. Along with this publication, 3D printed files can also be found at the National Institute of Health (NIH) 3D print exchange repository (https://3dprint.nih.gov/users/cto-laboratory) as well as at our laboratory website (http://www.bourginelab.com/).

### 5.5 Bioreactor components disinfection and assembly

For disinfection, the PLA-printed parts of the bioreactor (See Figure 1A; Supplementary Figure S1A) were immersed for a minimum of 30 min inside a solution of 70% Isopropanol prior to use. Before assembly, the parts were dried inside a sterile ventilated hood for 5 min. All other components of the bioreactors (c.f. Tubing and silicone O-ring) were sterilized by autoclaving. The assembly of the bioreactor and media change was carried out as previously described (Dupard and Bourgine, 2021) under a sterile hood; for natural collagen I (Col1), a 6 mm diameter scaffold (BD, Avitene Ultrafoam) was used; for crosslinked collagen (CrL-Col1), a 6–12 mm diameter ZimmerPatch Collagen sponge (ZimmerBiomet #0101Z) was used as scaffolding material. The infuse/withdraw PHD ULTRA™ syringe pump from Harvard Apparatus was plugged to the bioreactor for the dynamic perfusion of the nutritive media. F or the commercially available components of the bioreactor displayed in Supplementary Figure S1A, the manufacturers, in relation to the numbering are the following: 5: Silicon O-ring (#11.2007.0728; NORMATEC^®^ O-ring; 13 × 2 mm); 6: Lower stage of the bioreactor chamber; 7: Filtropur S plus .2 µm (#83.1826.102, Sarsted); 8: Female luer thread (#FTLL055-6,005, Nordson Medical); 9 and 9’: Silicon tubing of respectively 16 and 30 cm length (#8060-0060 Nalgene^®^ 50, ThermoFisher; inner diameter of 1/4 in); 10: Male luer lock (#MTLL055-6005, Nordson Medical); 11: Discofix^®^ C safeflow closed system stopcocks with valves (#16494CSF B. Braun) with BD PureHub™ disinfecting caps (#306596, Becton Dickinson); 12: Female Luer Lug (#FTL210-6,005, Nordson Medical); 13: Silicon tubing of 10 cm length (#8060-0020 Nalgene^®^ 50, ThermoFisher; inner diameter of 1/16 in); 14: Male luer lock (#MTLL220-6005, Nordson Medical).

### 5.6 3D printed PLA parts bacterial and mycoplasma contamination analysis

For contamination with mycoplasma, three chambers were left in culture with MSOD cells for 1 week. Ultimately, a 5 mL media sample was used for mycoplasma contamination assessment by the MycoplasmaCheck service from Eurofins Genomics. The performance of disinfection was evaluated after overnight incubation of 3D printed components in Lysogeny Broth (LB) medium at 37°C under 130 rpm shaking. Disinfection included 30 min exposure to UV light cabinet (#15572496, Fisher Scientific), or 30 min immersed in 70% isopropanol, controls consisted of immersion for 30 min in PBS. Bacterial growth was quantified through OD600 using the NanoDrop 2,000c from ThermoFisher.

### 5.7 Airtightness assessment of assembled chambers

Assembled chambers were occluded on one end and a syringe was plugged at the other end. To assess for airtightness the chamber was immersed in water and a gradual pressure of 1, 2 and 3 bar was applied through the syringe. If no air bubbles leaking from the chamber were observed, the chamber was considered airtight and fit for use in cell culture.

### 5.8 Human mesenchymal stromal cells 3D culture

5 million MSOD cells were suspended in 7 mL of CM for 3D perfusion and in 35 µL of CM for 3D static culture. Media change in perfusion settings was performed as previously described (Dupard and Bourgine, 2021) every 3 day. For static 3D culture, a 2 mL media change was carried out similarly. For static 3D culture, seeding was perfomed by capilarity on scaffolds placed in 12 well-plates coated with 1% agarose. For 6 mm scaffolds in perfusion culture, a first overnight infuse/withdraw perfusion cycle speed of 2.8 mL/min with displacement goal at 2 mL allowed dynamic cell seeding on the scaffold; for the rest of the 3D culture the infuse/withdraw perfusion cycle speed was lower at .28 mL/min. Moreover, speed was adjusted for different scaffold diameter sizes to ensure equivalent shear stress and fluid dynamics across conditions: 4.95 mL/min and .495 mL/min for 8 mm; 7.75 mL/min and .775 mL/min for 10 mm; 11.2 mL/min and 1.12 mL/min for 12 mm scaffolds.

### 5.9 MTT analysis

Scaffolds retrieved from the culture chamber were washed twice in pre-warmed PBS and incubated for 2 h at 37°C and 5% CO2 in DMEM (no phenol; Gibco #A1443001) with 50 μg/mL Thiazolyl Blue Tetrazolium Bromide (Sigma #M5655). Scaffolds were rinsed twice with pre-warmed PBS and cut in half in the median plane prior to imaging.

### 5.10 Cell proliferation assays in bioreactor perfused scaffold by CyQuant

For cell number determination, scaffolds were washed twice in pre-warmed PBS before being submerged in a digestion solution containing 1 mg/mL of Proteinase K (Sigma #P2308), 10 μg/mL of Pepstatin A (Sigma #P5318), 1 mM EDTA (Sigma #03690) and 1 mM of Iodoacetamide (Sigma #I6125) in a Tris buffer (Sigma #T5912) pH 7.6. The digestion was carried out overnight before being used for CyQuant™ Cell Proliferation Assay (Invitrogen #C7026) according to the manufacturer’s protocol. For comparison, 5 million MSOD cells were subjected to the same conditions and used for the determination of the ratio between DNA content measured by CyQuant to cell number.

### 5.11 Bioreactor reusability and BCA assay for post-culture adsorbed protein

To ascertain the reusability of the bioreactor, 3 sets of printed components were reused up to 4 times for 3 days of perfusion culture with 05 million MSOD cells seeded in 6 mm Col1 scaffolds. Between each experimental round, printed components were washed overnight in a cold water bath with a sodium lauryl sulfate (SLS)-based soap (Tork #420701), and then rinsed abundantly under running cold water. At the end of culture, the cell number was determined by CyQuant^TM^ assay as described above. To quantify the level of protein adsorbed on the bioreactor printed components after culture, and after washing, we used 8 mm scaffold holders used in perfusion culture for 5 days with 2 million MSOD cells seeded on 8 mm Col1 scaffolds. Samples consisted of scaffold holders dipped in 3 × 2 mL of PBS (“Before cleaning”); washed overnight with SLS-based soap, dried and left 30 min in 70% isopropanol (“After cleaning”), and new scaffold holders (“Freshly printed”). Proteins adsorbed were detached and solubilized by sonication for 10 min in 2 mL of RIPA buffer (Sigma #R0278). Solubilized proteins were then quantified using the bicinchoninic acid assay (BCA) according to the manufacturer’s protocol (Merck Millipore #71285-3). Absorbance at 562 nm was measured with the SPECTROstar Nano from BMG LabTech.

### 5.12 Mesenchymal-hematopoietic 3D coculture in perfusion bioreactor

3D coculture was performed as previously described (Dupard and Bourgine, 2021). Briefly, 35.000 UCB-CD34^+^ in CoM were injected in each perfusion bioreactor. 5 mL of media per bioreactor was changed every 2 days for 1 week, with re-injection in the system of the cellular populations retrieved from the withdrawn medium.

### 5.13 Sample preparation for flow cytometry

For 2D coculture, single-cell suspension was prepared by washing with PBS, followed by a 10 min digestion at 37°C in Trypsin-EDTA (.05%; Life Technology #25300054) supplemented with DNase I (.25 mg/mL; Thermofisher #10700595). Digestion was stopped by the addition of an equal volume of CoM. Cells were then resuspended in FACS buffer composed of PBS with 2% FBS and 1 mM EDTA and passed through a 40 µm nylon mesh. For 3D culture, PBS washes and trypsin digestion were carried out similarly under perfusion. All liquids were collected from the scaffold in a 50 mL tube and resuspended in FACS buffer. All samples were analyzed with an LSR Fortessa flow cytometer (BD Biosciences). Sytox Green (ThermoFisher #R37168) was used according to the manufacturer’s protocol and allowed the exclusion of both MSOD and dead cells from the analysis. Monoclonal antibodies against human CD34 (APC; 20:100; Clone: 581, BD #55824) and CD45ra (AF700; 10:100; BD #560673) allow the discrimination between committed (CD34^+^CD45ra+) and stem (CD34^+^CD45ra-) hematopoietic subpopulations. The committed population was further analysis for myeloid-lineage commitment with the expression of CD33 (BV650; 3:100; Biolegend #351904) surface marker (Myeloid-Primed: CD34^+^CD45ra-CD33^+^CD10^−^); while CD10 (BV421; 5:100; Clone: HI10a; Biolegend #312218) identified lymphoid lineage commitment (Lymphoid-Primed: CD34^+^CD45ra-CD33^−^CD10^+^). The stem cells compartment was further characterized with antibodies directed against EPCR (PE; 5:100; Biolegend #351904) and CD90 (PE-Cy7; 10:100; Clone: 5E10; Biolegend #561558) as previously described for *in vitro* hematopoietic culture76,77. Phenotypic hematopoietic stem cells (HSCs) were identified with the gating CD34^+^CD45ra-CD90+EPCR+; Multipotent progenitors population were defined as CD34^+^CD45ra- and further differentiated based on CD90 and EPCR expression (CD90+EPCR- and CD90-EPCR+). Samples were analyzed with FlowJo (version 10.7, BD Biosciences).

### 5.14 Immunofluorescence staining

Scaffolds used for imaging were fixed overnight in fresh 4% Paraformaldehyde at 4°C. Fixed samples were then washed in PBS and embedded in a 4% Low gelling Temperature Agarose (#A9414, Sigma). Longitudinal 100 µm thick sections in the median plane of the scaffold were then cut using a 7,000 smz vibratome with stainless steel at 50 Hz frequency, 1.5 mm amplitude, and .05 mm/s speed. For MSOD distribution in the scaffold, as the green fluorescence protein is constitutively expressed, no antibody staining was necessary. Tubulin was detected using the primary Rat anti-Tubulin (1:1,000; Abcam #GR3208838-5) and the secondary antibody Donkey anti-Rat (Cy3; 1:200; Sigma #SAB4600131). For CD45, detection was performed with the primary Mouse anti-CD45 (1:100; Ebiosciences #14-0459-82) and the secondary antibody Donkey anti-Mouse (CF633; 1:200; Jackson ImmunoResearch #712-165-150). All sections were mounted with Prolong™ Glass Antifade (ThermoFisher #P36982). Image acquisition was done with a Stellaris 5 confocal microscope from Leica.

### 5.15 Scanning electron microscopy (SEM) and pore size frequency

CrL-Col1 scaffold was mounted on a 12.5 mm aluminum stub. The scaffold was then sputtered with 10 nm Au/Pd (80/20) in a Quorum Q150 T ES turbo pumped sputter coater and examined in a Jeol JSM-7800 F FEG-SEM. Acquired micrographs of the top layer of the scaffold were used to determine pore diameters frequency *via* ImageJ (version 1.53f51). 127 pores were included in the analysis.

### 5.16 Statistical analysis

Statistical analysis was performed with R (version 4.1.3). Unless otherwise specified in figure legends, unpaired t-tests were used throughout the manuscript, with prior validation of test assumptions.

## Data Availability

The original contributions presented in the study are included in the article/Supplementary Material, further inquiries can be directed to the corresponding author.

## Supplementary material

Supplementary Figure S1
**(A)** Cross-section diagram revealing the primary components of the 3D printed perfusion bioreactor (left). 1: Representation of the alternating perfusion of culture media (blue) through the bioreactor; 2: Upper stage of the bioreactor chamber; 3: Collagen I (Col1) scaffold; 4: Scaffold holder; 5: Silicon O-ring; 6: Lower stage of the bioreactor chamber. Assembled 3D perfusion bioreactor; 7: Filtropur S plus 0.2 µm; 8: Female luer; 9 and 9’: Silicon tubing of respectively 16 and 30 cm length; 10: Male luer; 11: Discofix® C safeflow closed system stopcocks with valves with BD PureHub™ disinfecting caps; 12: Female Luer Lug; 13: Silicon tubing of 10 cm length; 14: Male luer lock. Components 2; 4 and 6 are 3D printed with polylactic acid (PLA). **(B)** The screw of the lower chamber stage fits threads of different commercially available 50 mL tubes, easing sample collection. **(C)** Component and assembly of the 3D printed bioreactor stand made from PLA.

Supplementary Figure S2
**(A)** The Mesenchymal Sword Of Damocles (MSOD) cells consist of an immortalized primary human mesenchymal stromal cell transfected with the human telomerase reverse transcriptase (hTERT) and inducible caspase 9 (iCaspase) death system. All systems are constitutively expressed. **(B)** Experimental diagram of the 2-week MSOD cells culture within the 3D printed perfusion bioreactor, using either the Col1 or CrL-Col1 scaffolds. Upon 1 week of culture, the use of a crosslinked material (CrL-Col1) avoided scaffold degradation. Col1: Collagen 1; CrL: Crosslinked. **(C)** Confocal microscopy of the 6 to 8 mm crosslinked (CrL)-Col1 scaffolding materials after 1 week of perfusion culture. The homogenous MSOD cells distribution at the top edge (dashed lines) of the median plane is evidenced. n = 3. Scale bar = 100 µm. eGFP: enhanced green fluorescent protein. **(D)** Scanning electron microscope micrographs of the top side of a 6 mm CrL-Col1 scaffold. Mechanical properties displayed in the table were gathered from (Chaudhury et al., 2012). **(E)** Frequency distribution of pore diameters at the top surface of a 6 mm CrL-Col1 scaffold. The median pore diameter is 81.49 µm (n = 127). (F) Bicinchoninic acid (BCA) quantification of proteins adsorbed on the scaffold holder after 5 days of MSOD culture, before and after cleaning, as well as freshly printed scaffold. ND = non-detected (< 20 µg).

Supplementary Figure S3
**(A)** Macroscopic image of a 6 mm crosslinked collagen scaffold 1 week post-3D mesenchymal-hematopoietic coculture (Niche condition). Scale bar = 1 mm **(B)** Experimental scheme of the 2D mesenchymal-hematopoietic coculture using MSOD and umbilical cord blood (UCB)-CD34+ cells. AA: Ascorbic Acid; TPO: Thrombopoietin; SCF: Stem Cell Factor; FLT3LG: Fms-related tyrosine kinase 3 ligand. **(C)** and **(D)** respectively shows hematopoietic committed populations and stem populations fold change from the UCB-CD34+ input (dotted line) after coculture. The “No stroma” condition refers to maintenance in culture without MSOD cells, as opposed to the “Stromal layer” condition. Unpaired t-test (*n* = 3). ns = *p*-value > 0.1. HSCs: Hematopoietic Stem Cells; MPPs: Multipotent Progenitors.
