# Supplementary figures and images for: Customizable 3D printed perfusion bioreactor for the engineering of stem cell microenvironments
Source: Front Bioeng Biotechnol. 2023 Jan 9;10:1081145. doi: 10.3389/fbioe.2022.1081145 (PMC9870251; doi:10.3389/fbioe.2022.1081145)

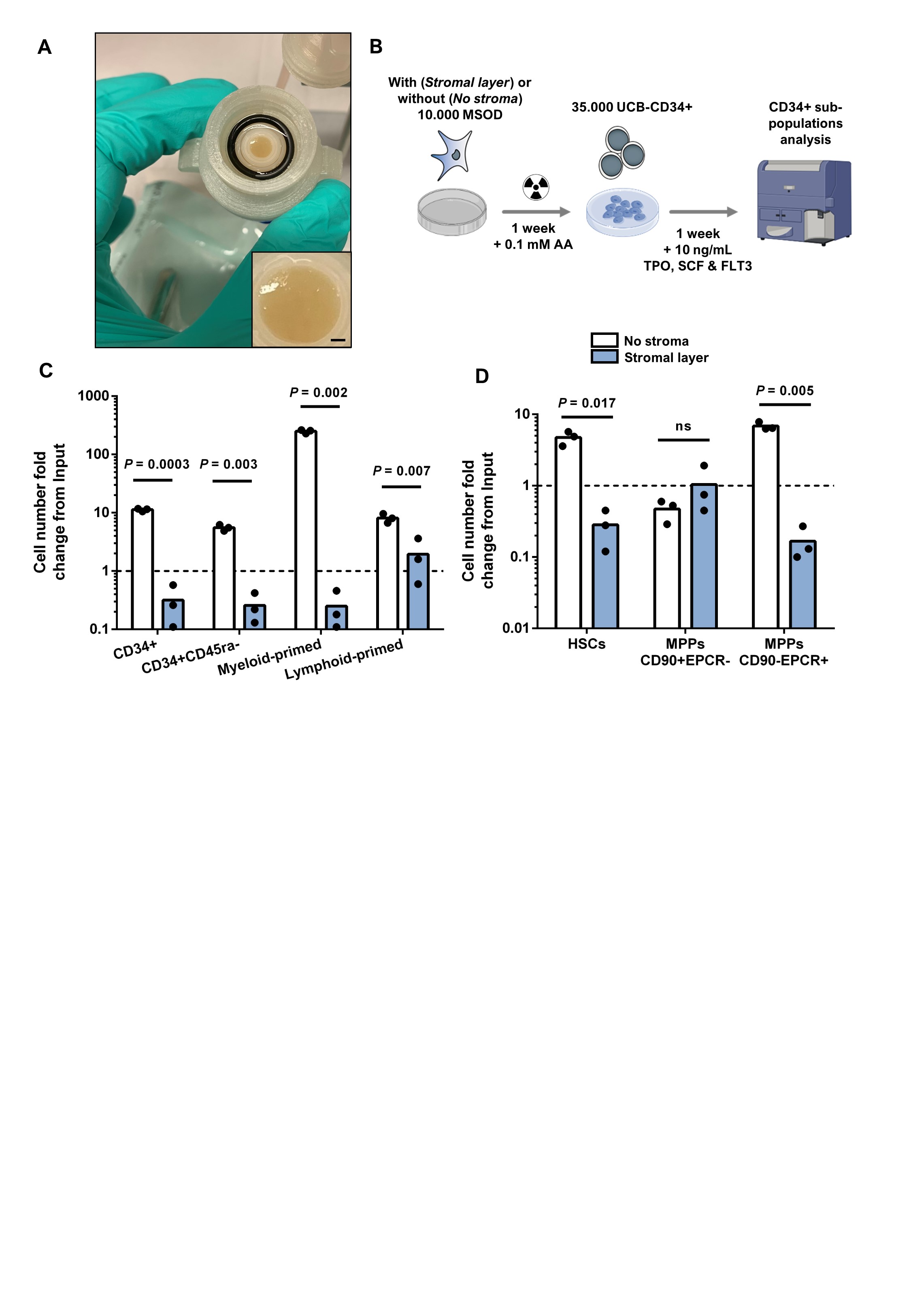

Supplement: Supplementary file 1 [file Image3.JPEG]

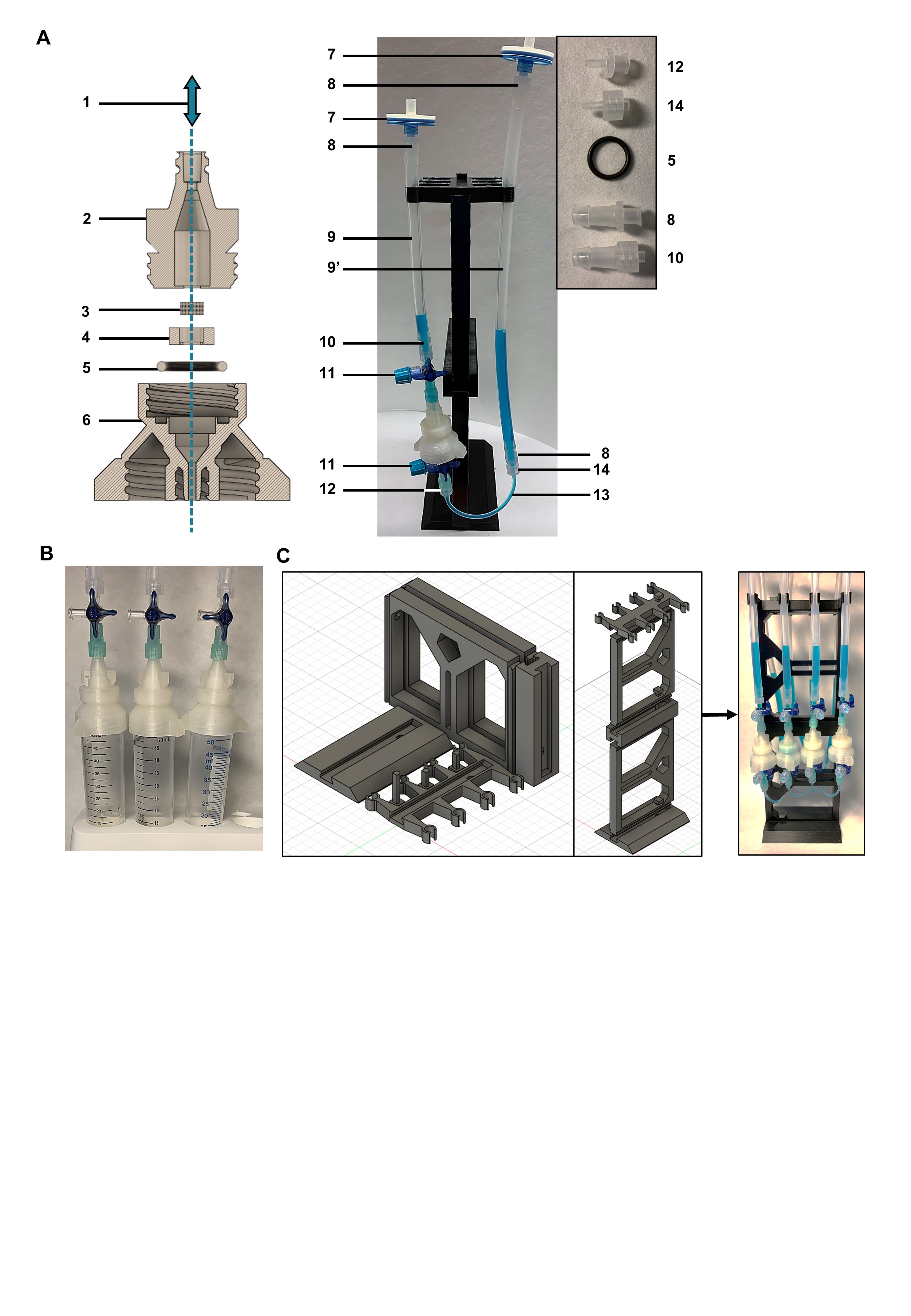

Supplement: Supplementary file 2 [file Image1.JPEG]

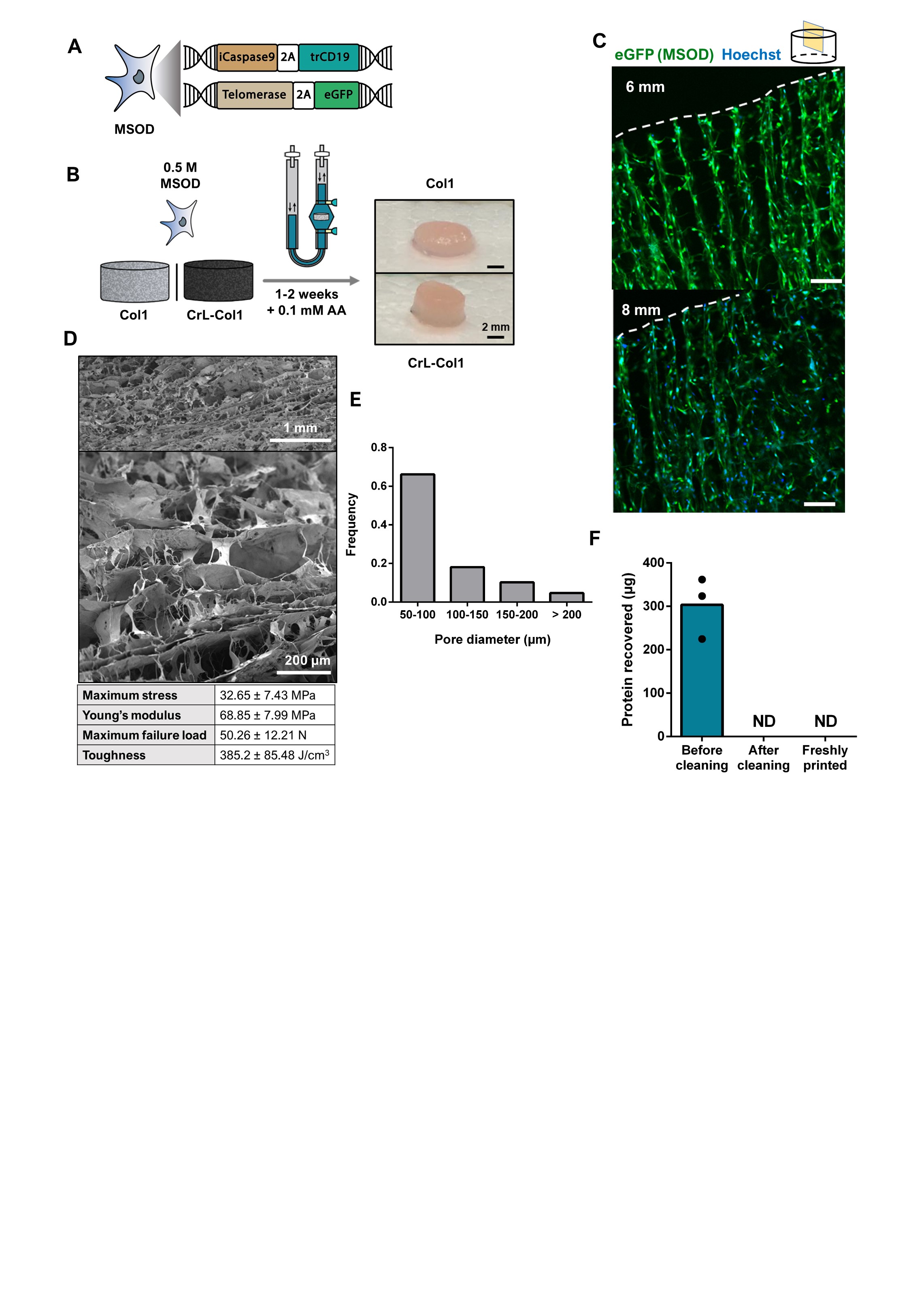

Supplement: Supplementary file 4 [file Image2.JPEG]
